# Self-confidence and knowledge of German ICU physicians in palliative care – a multicentre prospective study

**DOI:** 10.1186/s12904-017-0244-6

**Published:** 2017-11-22

**Authors:** Veronika Krautheim, Andrea Schmitz, Gesine Benze, Thomas Standl, Christine Schiessl, Wolfgang Waldeyer, Alexander Hapfelmeier, Eberhard F. Kochs, Gerhard Schneider, Klaus J. Wagner, Christian M. Schulz

**Affiliations:** 1 Klinik für Anaesthesiologie, Klinikum rechts der Isar, Technische Universität München, Ismaninger Straße 22, 81675 Munich, Germany; 2 Interdisziplinäres Zentrum für Palliativmedizin, Medizinische Fakultät Heinrich Heine Universität Düsseldorf, Düsseldorf, Germany; 3 Klinik für Palliativmedizin, Universitätsmedizin Göttingen, Göttingen, Germany; 4 Klinik für Anaesthesiologie, Operative Intensiv- und Palliativmedizin, Städtisches Klinikum Solingen, Solingen, Germany; 5 Algesiologikum München, Munich, Germany; 6 Institut für Medizinische Statistik und Epidemiologie, Klinikum rechts der Isar, Technische Universität München, Munich, Germany; 7 Klinik für Anaesthesiologie, Helios Universitätsklinikum Wuppertal, Wuppertal, Germany

**Keywords:** Knowledge, Palliative care, Self-confidence, Critical care, Gender

## Abstract

**Background:**

Little is known about ICU physicians’ self-confidence and knowledge related to palliative care. Our objective was to investigate self-confidence and knowledge of German ICU physicians related to palliative care, and to assess the impact of work experience, gender, specialty and additional certifications in pain or palliative medicine.

**Methods:**

In a multicentre prospective observational study ICU physicians of ten hospitals were asked to rate their self-confidence and to complete a multiple choice questionnaire for the assessment of knowledge. Beyond descriptive statistics and non-parametric tests for group comparisons, linear regression analysis was used to assess the impact of independent variable on self-confidence and knowledge. Spearman‘s rank test was calculated.

**Results:**

55% of answers in the knowledge test were correct and more than half of the participants rated themselves as “rather confident” or “confident”. Linear regression analysis revealed that an additional certificate in either pain or palliative medicine significantly increased both knowledge and self-confidence, but only 15 out of 137 participants had at least one of those certificates. Relation between self-confidence and the results of the knowledge test was weak (*r* = 0.270 in female) and very weak (*r* = −0.007 in male).

**Conclusions:**

Although the questionnaire needs improvement according to the item analysis, it appears that, with respect to palliative care, ICU Physicians’ self-confidence is not related to their knowledge. An additional certificate in either pain or palliative medicine was positively correlated to both self-confidence and knowledge. However, only a minority of the participants were qualified through such a certificate.

**Electronic supplementary material:**

The online version of this article (10.1186/s12904-017-0244-6) contains supplementary material, which is available to authorized users.

## Background

While intensive care medicine is traditionally committed to treat life-threatening organ dysfunction and to restore the patients’ health, palliative care focuses on quality of life in patients suffering from life-limiting illnesses at an advanced stage and also in deceasing patients and their relatives. The symptoms palliative and critical care patients suffer from widely overlap (e.g. pain, dyspnoea, delirium, disorientation, anxiety, nausea and vomiting), each of them requiring a specific treatment [1]. The need for palliative care on ICUs has increasingly been acknowledged in the past decade [2]. When palliative care competences are to be implemented in daily care, it is of importance to consider whether the policy of the respective ICU is based on an integrative model or a consultant model [3]. In an integrative model, ICU physicians have to possess palliative care competences; in contrast, in a consultant model these competencies are located outside the ICU, but ICU physicians have to initiate the referral following specific algorithms [3, 4]. Recently, it has been shown that integrating palliative care physicians into critical care significantly decreased ICU length of stay for referred patients without increasing mortality [5]. Nevertheless, another recent study identified a lack of competence in palliative care to contribute to futile life sustaining treatment in intensive care medicine [6].

In 2004, the German government recognized the need for improved palliative care [7]. Consecutively, in 2009, the Medical Licensure Act in Germany was adapted to improve palliative care and since then, palliative care is introduced for the medical students as a cross-sectional subject with obligatory examination [8]. Based on this novelty, the first students were licensed in 2014 [9]. But physicians currently working on intensive care units (ICUs) did not benefit from a curriculum on palliative medicine so far. However, there may be additional factors which may positively impact their self-confidence and knowledge related to palliative care such as work experience, [10, 11] additional certifications in either pain or palliative medicine, their medical background (specialty) [10, 11] or, possibly, gender [10–13]. Standardized questionnaires for this setting do not exist.

Therefore, this study aimed to investigate the current ICU physicians’ self-confidence and knowledge related to palliative care in an exploratory manner. For both, self-confidence and knowledge, a questionnaire was developed. Then, we analysed whether the results were associated with the participants’ additional certifications in either pain or palliative medicine, the overall amount of time they worked on ICU, their gender and professional specialties. Furthermore, results from the questionnaires’ item analysis are provided. Finally, we report the correlation between self-confidence and knowledge.

## Methods

### Study design

Between October 2012 and November 2013, in a multicentre prospective observational study, physicians who worked on ICUs in ten different hospitals in Germany were asked to participate unless their ICU work experience was of 1 month or less. The ICUs were selected by convenience sampling. In each of the participating ICU, the team had the possibility to consult a specialized palliative care team localized elsewhere in the respective hospital (consultation model). The study was approved by the Ethics Committee of the Faculty of Medicine of the Technische Universität München (N° 5314/12, March 5th 2012). The participants and heads of the involved departments provided written informed consent.

### Setting and participants

One hundred thirty seven out of 192 physicians agreed to participate (71.4%). The participants were from fifteen different ICUs of ten hospitals (4 academic teaching hospitals and six university hospitals). Demographic data about the participants is presented in Table 1. Only 15 out of 137 had an additional certificate in either pain (*n* = 9) and/or palliative medicine (*n* = 8, 2 participants had both certificates) (CPoP). Of those, 11 were senior consultants which is less than one third of all included senior consultants. Practically all certified participants were anaesthesiologists (*n* = 13).

**Table 1:** Demographic data

Age (mean ± SD)		37.0 ± 7.1
Sex w / m		53 (38.7%) / 84 (61.3%)
Specialty	Anaesthesiology	94 (71.8%)
	Operative	12 (9.2%)
	Non-operative	25 (19.1%)
Position	Resident	66 (48.2%)
	Consultant	33 (24.1%)
	Senior Consultant	38 (27.7%)
Overall work experience in years (median and IQR)		7.0 [4.0–12.0]
ICU work experience in years (median and IQR)		1.5 [0.5–3.0]
Certificate in pain medicine yes / no		9 (6.6%) / 128 (93.4%)
Certificate in palliative care yes / no		8 (5.8%) / 129 (94.2%)

Demographic data about the participating physicians. IQR interquartile range

### The standardized questionnaire

An extensive search for an adequate questionnaire revealed that validated tools were available only for nurses [14, 15] but not for ICU physicians. Furthermore, as some of the content was not related to the ICU setting and as this process itself would have required a validation, we decided to develop a new questionnaire for the purposes of this study in an iterative approach. First, based on the official catalogue of learning objectives for medical students from the German Society of Palliative Medicine, [16] an expert round^1^ identified the themes considered relevant for ICU physicians based on the modified Medical Licensure Act [8]. In a second step, the selection and weighting of themes were revised based on a selective literature research (Additional file 1: Appendix 1).

The resulting questionnaire consisted of three parts. The first part evaluated demographic data (age, sex, specialty, time spent on ICU and overall work experience in years, additional certification in either palliative care or pain medicine). Due to the low number of physicians certified in either pain or palliative medicine, this group was not analysed separately. The second part (Additional file 2: Appendix 2) assessed the physicians’ self-confidence related to palliative care. For this purpose, ten questions were posed each covering one theme. Answers were provided on a four-point Likert-scale ranging from “not confident” (1) and “rather not confident” (2) to “rather confident” (3) and “confident” (4). In order to objectively evaluate the participants’ knowledge, the third part consisted of 20 multiple choice questions. Each question required a correct answer among five choices. The correct answers were defined on the basis of the World Health Organization definition of palliative care [17] and based on the literature provided in Additional file 1: Appendix 1. One to four multiple choice questions were drafted per topic, according to their weighting (Additional file 1: Appendix 1). In an iterative approach, the multiple choice questions were pilot-tested in 18 test persons consisting of final year medical students, nurses, paramedics, residents and consultants with the aim of evaluating item difficulty and comprehensibleness. Based thereupon, the expert round designed the final version (Additional file 3: Appendix 3)*.*

In each hospital a local investigator asked all the physicians working on ICU to participate. When the questionnaire was administered, the participants were under supervision of a local investigator in order to avoid the consultation of external additives.With the objective of avoiding the self-confidence to be biased by the multiple choice questions, the participants had first to provide the assessment of their self-confidence and second, to answer the multiple choice questions. The participants were not allowed to page back and re-edit their answers. This was ensured by an independent investigator of the respective hospital. If the participant made no choice, the wrong choice or more than one choice, the answer was considered incorrect.

### Statistics

Results from the self-confidence and knowledge questionnaires were considered to be the dependent variables. Presence or absence of a CPoP, work experience, medical specialty and gender were independent variables. Corresponding hypothesis testing for group comparisons was performed by Mann-Whitney-U tests and Friedman rank sum tests. Multivariable analysis by linear regression analysis was used for adjusted effect estimation and hypothesis testing. For item analysis regarding self-confidence, missing responses, skew and % scores of the floor score “not confident” and ceiling score “confident” are reported [18]. For the knowledge test, item difficulty is provided as *p*-values, item discrimination as corrected point-biserial coefficients, and internal consistency as Cronbach’s alpha [19]. Correlation analyses based on Spearman’s rank correlation coefficient. Results of descriptive statistics of normally and non-normally distributed data are given as mean ± standard deviation and median (interquartile range; IQR), respectively. The distribution of qualitative data is presented by absolute and relative frequencies. All statistical tests were performed on exploratory two-sided 5% significance levels. Computations were performed with SPSS (SPSS Statistics for Windows, Version 23.0. Armonk, NY).

## Results

### Participants’ self-confidence

More than half of the participants felt “rather confident” or “confident” (median 2.7 [2.3–3.1]). Basics of palliative care and euthanasia were themes with low self-confidence (Table 2). An additional CPoP and ICU work experience was associated with significantly higher scores in self-confidence. Females tended to have a slightly lower self-confidence though not statistically significant (Table 3).

**Table 2:** Self-Confidence and knowledge related to topics

Topic		Self-confidence		Knowledge
		“Confident” and “Rather confident”	“Rather not confident” and “Not confident”	Percentage of correct answers
Basics of palliative care		32,4%	67,6%	64.2%
Symptom control	pain	78,7%	21,3%	44.4%
	dyspnoea	81,6%	18,4%	72.1%
	nausea and vomiting	89,7%	10,3%	50.0%
	delirium	57,4%	42,6%	52.9%
	the dying patient	55,1%	44,9%	43.4%
Ways of drug administration		60,7%	39,3%	20.6%
Communication with the patient		85,8%	41,2%	77.2%
Considering the patient’s wishes		76.5%	23.5%	83.9%
Euthanasia		46,3%	53,7%	55.2%

Self-confidence and knowledge according to topics. In Germany, “considering the patient’s wishes” is a hierarchical step-wise approach driven by the obligation to respect the patient’s autonomy. The wishes of an awake patient is mandatory. If the patient is not awake, wishes that eventually have been declared in written form and that account for the present situation have to be considered. If such a document does not exist, the patient’s presumable wishes has to be evaluated with him or her relatives based on earlier oral statements of the patient. If this is not possible (e.g. due to missing statements / relatives), medically indicated procedures have to be conducted.

In Germany, only passive euthanasia is legal. Passive euthanasia is defined as either the renunciation of life prolonging procedures or ending life prolonging procedures. It aims to not intentionally prolongate the process of dying and may include the initiation of a treatment (e.g. morphine) to relieve symptoms (pain, dyspnea) even if time until death is shortened

**Table 3:** Self-Confidence and knowledge according to gender, specialty, additional certificates and work experience

		Self-confidence (median [IQR])		Knowledge test (median [IQR])	
			*p*-value		*p*-value
Gender	Male	2.8 [2.4–3.2]	0.080	55% [49–60]	0.741
	Female	2.6 [2.3–3.0]		55% [45–65]	
Specialty	Anaesthesia	2.7 [2.3–3.1]	0.205	55% [46–64]	0.240
	other operative	2.5 [2.3–2.7]		53% [41–55]	
	other non-operative	2.8 [2.4–3.0]		57% [46–60]	
CPoP	no	2.6 [2.3–3.0]	< 0.001*	55% [45–60]	0.004*
	yes	3.4 [3.0–3.6]		60% [55–75]	
		Spearman Rho		Spearman Rho	
ICU work experience		0.499	< 0.001*	0.117	0.178

Results of participants’ self-confidence and knowledge according to gender, specialty, an additional certificate in pain or palliative medicine (CPoP) and ICU work experience. Results are given as median with interquartile range (IQR). Spearman’s rank correlation coefficient is provided as Spearman Rho. Asterics indicate statistical significance

Linear regression analysis showed a significant relation of self-confidence to the presence of a CPOP (mean increase 0.63 [95%-CI 0.36 to 0.90], *p* < 0.001) and ICU work experience (mean increase by 10 years of ICU work experience 0.43 [95%-CI 0.19 to 0.66], *p* < 0.001) but not gender (mean increase if participant was male 0.11 [95%-CI 0.06 to 0.28], *p* = 0.195).

Item analysis revealed a skew ranging between −0.549 and 0.445. The highest percentage was 27% for the ceiling score “confident” and 12% for the floor score “not confident”.

### Participants’ knowledge

The median [IQR] of percentage of correct answers in the objective knowledge test was 55 [45–60]. *Considering the patient’s wishes*, the *communication with the patient* and *controlling the symptom of dyspnoea* were the topics with the highest percentage of correct answers. In contrast, *ways of drug administration*, *controlling pain* and *symptoms in the dying patient* were the themes with the lowest percentage of correct answers (Table 2). An additional CPoP was associated with significantly higher scores. The participants’ ICU work experience, however, was not correlated with knowledge. Knowledge did not differ with respect to gender (Table 3).

Linear regression analysis revealed that an additional CPoP significantly increased the correct answers by on average 10.4% (95%-CI 3.7 to 17.1, *p* = 0.003). ICU work experience (mean increase of 3.8 [95%-CI 2.1 to 9.78], *p* = 0.203) and gender showed (mean increase if gender was male −1.1 [95%-CI -5.3 to 3.1], *p* = 0.595) no significant relation.

Knowledge test item analysis revealed a Cronbach’s alpha of 0.504. Their median *p*-value was 0.60 (interquartile range 0.22 to 0.85), which means that more than half of the items were very easy or very difficult. Median corrected point-biserial discrimination index was 0.15 (IQR 0.12–0.20).

### Correlation of participants’ self-confidence and knowledge

Overall, correlation between self-confidence and knowledge was poor (*r* = 0.104, *p* = 0.230). For example, 88.2% of the participants felt “confident” or “rather confident” in controlling the symptoms nausea and vomiting. However, in this topic only 50% of answers were correct (Table 2). Accordingly, linear regression analysis did not show an impact of knowledge on self-confidence (mean increase by percentage of correct answers 0.001 [95%-CI -0.008 to 0.006], *p* = 0.761).

**Fig. 1:**
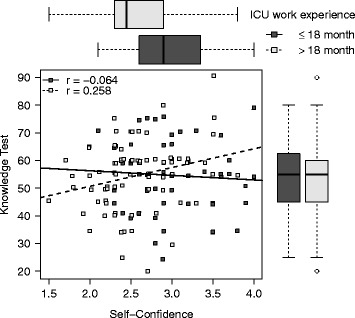
Self-confidence related to knowledge, presented for female and male participants separately. Spearman’s rank correlation coefficients are provided as r, boxplots represent median, interquartile range and end points

**Fig. 2:**
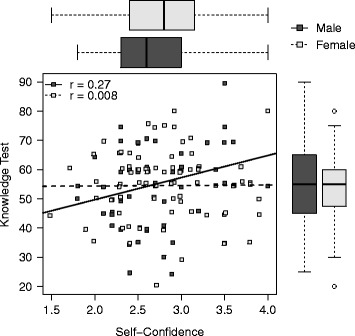
Self-confidence related to knowledge for participants with more or less than 18 months of ICU experience. Spearman’s rank correlation coefficients are provided as r, boxplots represent median, interquartile range and end points

Weak and very weak correlations between self-confidence and knowledge were found for female (*r* = 0.270, *p* = 0.050) and male participants (*r* = −0.007, *p* = 0.945) (Fig. 1). The more experienced ICU physicians (>1.5 years) showed a better correlation between self-confidence and knowledge (*r* = 0.258, *p* = 0.026) than the less experienced (<1.5 years) participants (*r* = −0.064, *p* = 0.627) (Fig. 2). Correlation increased slightly from those without CPoP (*r* = −0.017, *p* = 0.855) to those with CPoP (*r* = 0.148, *p* = 0.600).

## Discussion

The main finding of the study is the contradictory results of the assessment of self-confidence with more than half or participants rating themselves as “confident” or “rather confident” on one hand, and only 55% of correct answers in the knowledge test on the other hand. This is reflected by the poor correlation (*r* = 0.104) between self-confidence and knowledge that was surprising to us. From the independent variables, only the presence of a CPoP was associated positively with both the participants’ knowledge and self-confidence.

There were two themes (basics in palliative care and euthanasia) with which more than half of the participants felt not or rather not confident (Table 2). Although weak, self-confidence showed a significant correlation with the results of knowledge testing in female but not in male. Similar results have been found in other studies where female participants provided lower self-assessments although they performed equivalently [12] or even better [13] than their male colleagues. Another study assessed self-rated knowledge in end-of-life care and did not find differences between women and men [10]. From the investigated independent variables only the presence of a CPoP and work experience was associated positively with the participants’ self-confidence. According to the results of the regression analysis, the effect of work experience, however, is very weak compared to the impact of the CPoP.

Themes with low percentage of correct answers in the MCQ were ways of drug administration, controlling pain and symptoms in the dying patient, which is not consistent with the themes with a low level of self-confidence (basics in palliative care and euthanasia). In most ICU patients, drug administration occurs primarily orally or intravenously. Most participants possibly felt these ways to be sufficient for accurate palliative care. Alternative ways of drug administration are rarely considered necessary. This may explain the low percentage of correct answers with respect to this topic. More than half of the answers were very difficult or very easy according to the *p*-values, suggesting that validity could be improved by revising these items.

Importantly, knowledge is not competence. The assessment of conscious knowledge, corresponding to Kirkpatrick’s level II, does not necessarily predict a physicians’ interplay with their clinical environment (Kirkpatrick’s level IV) [20]. That is, competence may result from experience-based intuitive and unconscious behaviour [21]. Therefore, MCQ results, although alarming, do not allow for direct conclusions on the participants’ ability to provide accurate palliative care in their respective work environment. In all investigated hospitals the ICU physicians had the opportunity to consult a palliative care team. Finally, as 1) MCQs serve for knowledge testing but not necessarily for the assessment of competence and 2) the frequency with which a palliative care team was consulted was not assessed, we cannot state the quality of palliative care in this sample.

Work experience was associated with self-confidence but not with knowledge (Table 3). We suggest that more experienced ICU physicians may have competence that is not reflected by the knowledge or tend to overestimate their abilities. Such an additional qualification was present in only 10% of all participants, increasing to 29% in senior consultants. This implies that less than one third of the leading ICU personnel had a CPoP. A similar gap has been described for communication: two studies reported differences between the physicians’ rating of their own communication skills compared to the patients’ perception of the physicians’ communication skills [22, 23].

There is hardly any literature evaluating knowledge and self-confidence related to palliative care. In a study involving physicians and nurses in an ICU setting, the need for improvement was found mainly for spiritual support for families, emotional support for clinicians, and education about palliative care [18]. Not surprisingly, another study found palliative care specialists and geriatrists to know more about the laws regulating the withholding/withdrawing life-sustaining treatment than other specialties [24]. In internal medicine residents, only clinical experience was found to be correlated positively to self-confidence and the authors conclude that increasing the experience in treating patients in end-of-life situations with appropriate supervision may result in improved competence [25]. Haematology/oncology fellows rated palliative care education within this fellowship to be inferior to general oncology and the authors identified the themes pain management, identifying the patients’ psychosocial needs and communication as promising goals for training [26]. Elsewhere, data are provided about palliative care knowledge and the impact of learning interventions for students [27] and nurses [28, 29] but the results cannot be transferred to ICU physicians.

Taken together, the studies related to palliative care do not consistently identify particular points of improvement. An important first step for ICU physicians is the transition from curative to palliative care in the individual patient [30]. This often proves to be rather difficult when faced with families’ resistance towards therapy ptions other than curative as well as their concerns physicians might give up on their relative [31]. Numerical rating scales, the Behaviour Pain Scale and the Respiratory Distress Observation Scale help to recognize and assess typical symptoms of the dying patient quickly [32]. Our data suggest that, beyond the implementation of rating scales, additional qualifications in palliative care beyond the regular curricula in ICU medicine enhance knowledge on one hand, and self-confidence on the other. In Germany, qualification in pain medicine requires a physician to complete an 80-h course, to have worked in pain medicine 1 year full-time with expert supervision and to have finished residency. To be certified in palliative care, a physician has to complete a 40-h course, and must have either worked 1 year in palliative care or, alternatively, complete a 120 h of case conferences and provide 20 extensive case reports, while having worked in direct patient care for at least 2 years. The results suggest that these qualifications are useful to enhance palliative care in the integrative model. An important next step is to investigate whether the introduction of the subject palliative care into the medical studies in Germany will influence self-confidence and knowledge once these students work as residents on ICUs.

Several limitations have to be considered. A point of concern is that the questionnaire did not undergo a prior formal validity testing prior the administration, and the *post-hoc* item analysis revealed that the MCQ may benefit from an adaption to the specific setting of ICUs. The item analysis identified items that were either too easy or too hard, which may impact the knowledge questionnaires’ validity. However, as ICU physicians trained in either pain or palliative medicine (as represented by the CPoP) performed better than non-trained ICU physicians, we suggest that the MCQ provides sufficient construct validity. This can be confirmed in future samples beyond the ICU setting. Although more than two thirds of the ICU personnel participated (71.4%), this study only reflects the situation in the participating ICUs and is therefore not necessarily representative for all German ICUs.

## Conclusions

Although there are limitations in the use of our questionnaire, it appears that, with respect to palliative care, ICU Physicians’ self-confidence is not related to their knowledge. An additional certificate in either pain or palliative medicine was positively correlated to both self-confidence and knowledge. However, only a minority of the participants were qualified through such a certificate. Future research should identify topics for goal-directed interventions to strengthen competence in palliative care in the ICU setting.

## Additional files

Additional file 1: Appendix 1. Topics, number of associated items and relevant literature. (DOCX 87 kb)

Additional file 2: Appendix 2. Questionnaire for the evaluation of the participants’ self-confidence related to palliative care medicine. (DOCX 88 kb)

Additional file 3: Appendix 3. Knowledge test. (DOCX 20 kb)

## Abbreviations

ÄApprO: Ärztliche Approbationsordnung
CPoP: Certificate in *either* pain *or* palliative medicine
ICU: Intensive care unit
IQR: Interquartile range

## Footnotes

1 (Prof. Dr. Friedemann Nauck, Past-President of the German Society of Palliative Medicine; Prof. Dr. med. Bernd Alt-Epping and Prof. Dr. Boris Zernikow, members of the Editorial board of the Zeitschrift für Palliativmedizin; Dr. Julia Wager, Centre of Pediatric Pain Therapy, University of Witten/Herdicke, and the following authors of this study KW, WW, VK)

## References

[1] Laugsand EA, Kaasa S, de Conno F, Hanks G, Klepstad P (2009). Research steering Committee of the E: intensity and treatment of symptoms in 3,030 palliative care patients: a cross-sectional survey of the EAPC research network. J Opioid Manag.

[2] Cook D, Rocker G (2014). Dying with dignity in the intensive care unit. N Engl J Med.

[3] Nelson JE, Bassett R, Boss RD, Brasel KJ, Campbell ML, Cortez TB, Curtis JR, Lustbader DR, Mulkerin C, Puntillo KA (2010). Models for structuring a clinical initiative to enhance palliative care in the intensive care unit: a report from the IPAL-ICU project (improving palliative care in the ICU). Crit Care Med.

[4] Campbell ML (2006). Palliative care consultation in the intensive care unit. Crit Care Med.

[5] Walker KA, Mayo RL, Camire LM, Kearney CD (2013). Effectiveness of integration of palliative medicine specialist services into the intensive care unit of a community teaching hospital. J Palliat Med.

[6] Jox RJ, Schaider A, Marckmann G, Borasio GD (2012). Medical futility at the end of life: the perspectives of intensive care and palliative care clinicians. J Med Ethics.

[7] Jaspers B, Schindler T: Stand der Palliativmedizin und Hospizarbeit in Deutschland und im Vergleich zu ausgewählten Staaten. Gutachten. Enquête-Kommission des Bundestages “Ethik und Recht der modernen Medizin” Berlin, November 2004.

[8] Elsner F. Das neue DGP-Curriculum für Studierende. Z Palliativmed. 2009;10(4):182.

[9] Weber M, Schmiedel S, Nauck F, Alt-Epping B (2011). Knowledge and attitude of final - year medical students in Germany towards palliative care - an interinstitutional questionnaire-based study. BMC Palliat Care.

[10] Bradley EH, Cramer LD, Bogardus ST, Kasl SV, Johnson-Hurzeler R, Horwitz SM (2002). Physicians’ ratings of their knowledge, attitudes, and end-of-life-care practices. Acad Med.

[11] Prem V, Karvannan H, Kumar SP, Karthikbabu S, Syed N, Sisodia V, Jaykumar S (2012). Study of Nurses’ knowledge about palliative care: a quantitative cross-sectional survey. Indian J Palliat Care.

[12] Minter RM, Gruppen LD, Napolitano KS, Gauger PG (2005). Gender differences in the self-assessment of surgical residents. Am J Surg.

[13] Colbert-Getz JM, Fleishman C, Jung J, Shilkofski N (2013). How do gender and anxiety affect students’ self-assessment and actual performance on a high-stakes clinical skills examination?. Acad Med.

[14] Pfister D, Muller M, Muller S, Kern M, Rolke R, Radbruch L (2011). Validation of the Bonn test for knowledge in palliative care (BPW). Schmerz.

[15] Nakazawa Y, Miyashita M, Morita T, Umeda M, Oyagi Y, Ogasawara T (2009). The palliative care knowledge test: reliability and validity of an instrument to measure palliative care knowledge among health professionals. Palliat Med.

[16] Palliativmedizin DGf: Curriculum: Grundlagen der Palliativmedizin. Gegenstandskatalog und Lernziele für Studierende der Medizin (2nd Edition). 2009.

[17] WHO Definition of Palliative Care. 2017. http://www.who.int/cancer/palliative/definition/en/. Accessed 29 May 2017.

[18] Ho LA, Engelberg RA, Curtis JR, Nelson J, Luce J, Ray DE, Levy MM (2011). Comparing clinician ratings of the quality of palliative care in the intensive care unit. Crit Care Med.

[19] Lei PW, Wu Q (2007). CTTITEM: SAS macro and SPSS syntax for classical item analysis. Behav Res Methods.

[20] Boet S, Bould MD, Fung L, Qosa H, Perrier L, Tavares W, Reeves S, Tricco AC (2014). Transfer of learning and patient outcome in simulated crisis resource management: a systematic review. Can J Anaesth.

[21] ten Cate O, Snell L, Carraccio C (2010). Medical competence: the interplay between individual ability and the health care environment. Med Teach.

[22] Tongue JR, Epps HR, Forese LL (2005). Communication skills. Instr Course Lect.

[23] Stewart MA (1995). Effective physician-patient communication and health outcomes: a review. CMAJ.

[24] Cartwright CM, White BP, Willmott L, Williams G, Parker MH (2016). Palliative care and other physicians’ knowledge, attitudes and practice relating to the law on withholding/withdrawing life-sustaining treatment: survey results. Palliat Med.

[25] Billings ME, Curtis JR, Engelberg RA (2009). Medicine residents’ self-perceived competence in end-of-life care. Acad Med.

[26] Buss MK, Lessen DS, Sullivan AM, Von Roenn J, Arnold RM, Block SD (2011). Hematology/oncology fellows’ training in palliative care: results of a national survey. Cancer.

[27] Gerlach C, Mai S, Schmidtmann I, Massen C, Reinholz U, Laufenberg-Feldmann R, Weber M (2015). Does interdisciplinary and multiprofessional undergraduate education increase students’ self-confidence and knowledge toward palliative care? Evaluation of an undergraduate curriculum design for palliative care at a German academic hospital. J Palliat Med.

[28] Shipman C, Burt J, Ream E, Beynon T, Richardson A, Addington-Hall J (2008). Improving district nurses’ confidence and knowledge in the principles and practice of palliative care. J Adv Nurs.

[29] Morita T, Fujimoto K, Imura C, Nanba M, Fukumoto N, Itoh T (2006). Self-reported practice, confidence, and knowledge about palliative care of nurses in a Japanese regional cancer center: longitudinal study after 1-year activity of palliative care team. Am J Hosp Palliat Care.

[30] Coombs MA, Addington-Hall J, Long-Sutehall T (2012). Challenges in transition from intervention to end of life care in intensive care: a qualitative study. Int J Nurs Stud.

[31] Karlekar M, Collier B, Parish A, Olson L, Elasy T (2014). Utilization and determinants of palliative care in the trauma intensive care unit: results of a national survey. Palliat Med.

[32] Puntillo K, Nelson JE, Weissman D, Curtis R, Weiss S, Frontera J, Gabriel M, Hays R, Lustbader D, Mosenthal A (2014). Palliative care in the ICU: relief of pain, dyspnea, and thirst--a report from the IPAL-ICU advisory board. Intensive Care Med.

