# Supplementary material for: Self-confidence and knowledge of German ICU physicians in palliative care – a multicentre prospective study
Source: BMC Palliat Care. 2017 Nov 22;16:57. doi: 10.1186/s12904-017-0244-6 (PMC5700543; doi:10.1186/s12904-017-0244-6)
Supplement: Supplementary file 1 — Appendix 1. Topics, number of associated items and relevant literature. (DOCX 87 kb) [file 12904_2017_244_MOESM1_ESM.docx]

**Additional file 1: Appendix 1**

| Theme | Number  of items | Literature |
| --- | --- | --- |
| Definition of Palliative Care Medicine | 1 | Klaschik E, Nauck F, Radbruch L, Sabatowski R (2000) Palliative medicine - definitions and principles. [Internist](http://www.ncbi.nlm.nih.gov/pubmed?term=palliative%20medicine%20definitions%20and%20principles.%202000&cmd=correctspelling##) 41: 606-611 |
| Availability of Palliative Care in Germany | 2 | Sabatowski R, Radbruch L, Nauck L, Loick G, Meuser T , Lehmann KA, Sabatowski R (2000) Über die Entwicklung palliativmedizinischer Einrichtungen in Deutschland. Palliativmedizin 1: 40-46 |
| Pain | 4 | Grond S, Radbruch L (1998) Weak opioids. Meta-analysis for the therapy of chronic pain. Schmerz 12: 142-155  Meuser T, Pietruck C, Radbruch L, Stute P, Lehmann KA, Grond S (2001) Symptoms during cancer pain treatment following WHO-guidelines: a longitudinal follow-up study of symptom prevalence, severity and aetiology. [Pain](http://www.ncbi.nlm.nih.gov/pubmed?term=Symptoms%20during%20cancer%20pain%20treatment%20following%20WHO-guidelines%3A%20a%20longitudinal%20follow-up%20study%20of%20symptom%20prevalence%2C%20severity%20and%20etiology.##): 93: 247-257 |
| Other symptoms requiring treatment | 4 | [Laugsand](http://www.ncbi.nlm.nih.gov/pubmed?term=%22Laugsand%20EA%22%5BAuthor%5D) EA (2009) Intensity and treatment of symptoms in 3030 palliative care patients: a cross-sectional survey of the EAPC Research Network. [J Opioid Manag](http://www.ncbi.nlm.nih.gov/pubmed/19344044##): 5: 11-21 |
| Different ways of drug administration | 1 | [Ripamonti](http://www.ncbi.nlm.nih.gov/pubmed/?term=Ripamonti%20C%5BAuthor%5D&cauthor=true&cauthor_uid=2045992) C, [Bruera EJ (1991)](http://www.ncbi.nlm.nih.gov/pubmed/?term=Bruera%20E%5BAuthor%5D&cauthor=true&cauthor_uid=2045992) Rectal, buccal, and sublingual narcotics for the management of cancer pain. [J. Palliat Care](http://www.ncbi.nlm.nih.gov/pubmed/2045992): 7: 30-5  [De Conno](http://www.ncbi.nlm.nih.gov/pubmed/?term=De%20Conno%20F%5BAuthor%5D&cauthor=true&cauthor_uid=7707099) F, [Ripamonti](http://www.ncbi.nlm.nih.gov/pubmed/?term=Ripamonti%20C%5BAuthor%5D&cauthor=true&cauthor_uid=7707099) C, [Saita](http://www.ncbi.nlm.nih.gov/pubmed/?term=Saita%20L%5BAuthor%5D&cauthor=true&cauthor_uid=7707099) L, [MacEachern](http://www.ncbi.nlm.nih.gov/pubmed/?term=MacEachern%20T%5BAuthor%5D&cauthor=true&cauthor_uid=7707099) T, [Hanson](http://www.ncbi.nlm.nih.gov/pubmed/?term=Hanson%20J%5BAuthor%5D&cauthor=true&cauthor_uid=7707099) J, [Bruera](http://www.ncbi.nlm.nih.gov/pubmed/?term=Bruera%20E%5BAuthor%5D&cauthor=true&cauthor_uid=7707099) EJ (1995) Role of rectal route in treating cancer pain: a randomized crossover clinical trial of oral versus rectal morphine administration in opioid-naive cancer patients with pain. [J Clin Oncol](http://www.ncbi.nlm.nih.gov/pubmed/7707099): 13: 1004-8  [Coyle](http://www.ncbi.nlm.nih.gov/pubmed/?term=Coyle%20N%5BAuthor%5D&cauthor=true&cauthor_uid=8003396) N, [Cherny](http://www.ncbi.nlm.nih.gov/pubmed/?term=Cherny%20NI%5BAuthor%5D&cauthor=true&cauthor_uid=8003396) NI, [Portenoy](http://www.ncbi.nlm.nih.gov/pubmed/?term=Portenoy%20RK%5BAuthor%5D&cauthor=true&cauthor_uid=8003396) RK (1994) Subcutaneous opioid infusions at home. [Oncology:](http://www.ncbi.nlm.nih.gov/pubmed/8003396) 8: 21-7; discussion 31-2, 37 |
| Liquid and nutrition in the dying patient | 2 | [Jox RJ](http://www.ncbi.nlm.nih.gov/pubmed?term=Jox%20RJ%5BAuthor%5D&cauthor=true&cauthor_uid=19682847), [Krebs M](http://www.ncbi.nlm.nih.gov/pubmed?term=Krebs%20M%5BAuthor%5D&cauthor=true&cauthor_uid=19682847), [Fegg M](http://www.ncbi.nlm.nih.gov/pubmed?term=Fegg%20M%5BAuthor%5D&cauthor=true&cauthor_uid=19682847), [Reiter-Theil S](http://www.ncbi.nlm.nih.gov/pubmed?term=Reiter-Theil%20S%5BAuthor%5D&cauthor=true&cauthor_uid=19682847), [Frey L](http://www.ncbi.nlm.nih.gov/pubmed?term=Frey%20L%5BAuthor%5D&cauthor=true&cauthor_uid=19682847), [Eisenmenger W](http://www.ncbi.nlm.nih.gov/pubmed?term=Eisenmenger%20W%5BAuthor%5D&cauthor=true&cauthor_uid=19682847), [Borasio GD](http://www.ncbi.nlm.nih.gov/pubmed?term=Borasio%20GD%5BAuthor%5D&cauthor=true&cauthor_uid=19682847) (2010) Limiting life-sustaining treatment in German intensive care units: a multiprofessional survey. [J Crit Care](http://www.ncbi.nlm.nih.gov/pubmed/19682847): 25: 413-9  Nauck F (2001) Symptom control in the terminal phase. Schmerz: 15: 362-369 |
| Terminal care | 2 | Klaschik E (2008) Sterbehilfe – Sterbebegleitung. Anaesthesist: 49: 420-426 |
| Respecting the patient’s will | 2 | Borasio GD, Heßler H-J, Wiesing U (2009) Patientenverfügungsgesetz. Dtsch Ärztebl: 106: A 1952–1957 |
| Communication | 2 | Frick E, Riedner C, Fegg MJ, Hauf S, Borasio GD (2006) A clinical interview assessing cancer patients’ spiritual needs and preferences. [Eur J Cancer Care](http://www.ncbi.nlm.nih.gov/pubmed?term=A%20clinical%20interview%20assessing%20cancer%20patients%E2%80%99%20spiritual%20needs%20and%20preferences##):15: 238-43  van Oorschot B, Neuderth S, Faller H, Flentje M (2008) Kommunikation mit Palliativpatienten - Erste Erfahrungen mit standardisierten Patienten im Rahmen der Interdisziplinären Onkologie. Palliativmedizin: 9: 120-125  Wasner M, Kaub-Wittmer D (2006) Kommunikation und psychosoziale Begleitung: Kernkompetenzen in der Palliativmedizin. DZO: 38: 174-177 |

Appendix 1: Topics, number of associated items and relevant literature
