# Supplementary material for: Self-confidence and knowledge of German ICU physicians in palliative care – a multicentre prospective study
Source: BMC Palliat Care. 2017 Nov 22;16:57. doi: 10.1186/s12904-017-0244-6 (PMC5700543; doi:10.1186/s12904-017-0244-6)
Supplement: Supplementary file 2 — Appendix 2. Questionnaire for the evaluation of the participants’ self-confidence related to palliative care medicine. (DOCX 88 kb) [file 12904_2017_244_MOESM2_ESM.docx]

# Additional file 2: Appendix 2

# Self-confidence

Please evaluate your level of confidence for each of the following scenarios relating to palliative medicine on a scale of 1 to 4 (1 =confident, 2 = rather confident, 3 = rather not confident, 4 = not confident).

|  | 1 | 2 | 3 | 4 |
| --- | --- | --- | --- | --- |
| 1. With respect to my knowledge of the basic principles of Palliative medicine, I consider myself… |  |  |  |  |
| 1. With respect to the management of pain in palliative care, I consider myself… |  |  |  |  |
| 1. With respect to the management of dyspnoea in palliative care, I consider myself… |  |  |  |  |
| 1. With respect to the management of nausea and vomiting in palliative care, I consider myself... |  |  |  |  |
| 1. With respect to the management of delirium, I consider myself... |  |  |  |  |
| 1. With respect to the management of a patient who is expected to die within the next 72 hours , I consider myself... |  |  |  |  |
| 1. With respect to my knowledge of different routes of medication delivery in palliative care, I consider myself... |  |  |  |  |
| 1. With respect to communicating with palliative care patients, I consider myself... |  |  |  |  |
| 1. With respect to consideration for and respect of the individual wishes of patients, I consider myself... |  |  |  |  |
| 1. With respect to my knowledge and understanding of the legal aspects of euthanasia and assisted dying, I consider myself… |  |  |  |  |

Appendix 2: Questionnaire for the evaluation of the participants’ self-confidence related to palliative care medicine.
