# Supplementary material for: Self-confidence and knowledge of German ICU physicians in palliative care – a multicentre prospective study
Source: BMC Palliat Care. 2017 Nov 22;16:57. doi: 10.1186/s12904-017-0244-6 (PMC5700543; doi:10.1186/s12904-017-0244-6)
Supplement: Supplementary file 3 — Appendix 3. Knowledge test. (DOCX 20 kb) [file 12904_2017_244_MOESM3_ESM.docx]

# Additional file 3: Appendix 3. Knowledge test

1. Which one of the following patients is the least suited for a palliative care referral, based on palliative medicine criteria:

- - 1. Status post second liver transplant (after failure of the initial liver transplant), as treatment for alcoholic liver cirrhosis and hepatorenal syndrome
    2. Status post laparotomy with small bowel resection due to ileus, on a background of ovarian carcinoma with ascites
    3. Status post vertebral body lesion resection due to metastatic breast carcinoma, complicated by a prolonged mechanical ventilation requirement due to pulmonary infection
    4. Status post second myocardial infarction with severe cardiac failure, on a background of COPD and end-stage renal disease requiring dialysis.
    5. Status post basal ganglia haemorrhage with right sided hemiparesis, on a background of Alzheimer’s dementia.

2. SAPV (spezialisierte ambulante Palliativversorgung, or home-based specialised palliative care) refers to:

- - 1. A fast home-based based palliative care service provided by a Hospice-type team.
    2. A additional, paid-for, comprehensive palliative care service provided by a general practitioner.
    3. A specialised home-based based palliative care service provided by appropriately trained personnel in addition to general practitioners and nurses.
    4. A fast home-based based palliative care service provided by trained emergency doctors.
    5. A specialised home-based based palliative care service provided by volunteers.

3. Please choose the most appropriate answer: a patient can be transferred to a palliative care ward when:

- - 1. The patient refuses interventions that would prolong his or her life.
    2. The relatives insist on the transfer, without the consent of the patient if needs be.
    3. The patient was transferred initially from the intensive care unit to a normal ward.
    4. The patient suffers from an advanced and incurable illness with distressing symptoms that cannot be adequately managed in the outpatient setting.
    5. The patient is eligible for discharge from the normal ward, but the relatives are not able to continue appropriate care at home.

4. Which statement relating to equipotential drug dosing is correct:

- - 1. Sufentanil 5 µg/h i.v. over 24 hours (120µg/24hours) is equivalent to approximately 90 mg piritramide i.v./24h
    2. A fentanil-patch 100 µg/h is equivalent to approximately 120 mg of morphine p.o./d
    3. Hydromorphone 16 mg p.o./d is equivalent to approximately 40 mg oxycodone p.o./d
    4. Tilidine 600 mg p.o./d is equivalent to approximately 30 mg piritramide i.v./d
    5. Oxycodone 30 mg p.o./d is equivalent to approximately 300 mg tramadol p.o./d

5. A patient with a prescription for 30-0-30 extended-release morphine PO requires PRN (as needed) analgesics 8 times per day for “break-through pain”. How would you write up his new daily prescription?

- - 1. 40-0-40 mg
    2. 50-0-50 mg
    3. 60-0-60 mg
    4. 70-0-70 mg
    5. 80-0-80 mg

6. A cancer patient receives morphine 90 mg p.o. daily in slow-release form. The patient is pain free and not overdosed. The patient however can currently no longer swallow, and requires the equivalent dose of morphine in subcutaneous form, 4 times per day (q.i.d.). How many milligrams per dose does the patient require?

- - 1. 2,5 mg
    2. 5,0 mg
    3. 7,5 mg
    4. 10 mg
    5. 20 mg

7. Which statement is most correct? The Total Pain Concept:

- - 1. Describes the worsening of pain in tumour patients shortly before death.
    2. Describes a state of pain in palliative care that is no longer manageable with medication.
    3. Comprises the biological, psychological, social and spiritual aspects of pain.
    4. Is always accompanied by severe physical pain.
    5. Describes an archaic, out of date pain management concept.

8. Which of the following is NOT a sensible treatment for reducing the stench of a foul-smelling ulcerating tumour?

- - 1. Ingestion of chlorophyll capsules
    2. Ingestion of charcoal tablets
    3. Topical application of activated charcoal dressings
    4. Topical application of a chlorophyll solution
    5. Topical application of sage tea

9. A 43 year old female patient is admitted to hospital suffering from ovarian cancer complicated by peritoneal metastasis, ascites and worsening dyspnoea (spO2 95%). You suspect pleural metastasis with effusion as the most likely cause. The patient is very restless, and a comprehensive history is not possible due to her severe dyspnoea. Which of the following is your first intervention?

- - 1. You inject the patient with intravenous morphine in 1 mg titrated boluses.
    2. You order a chest x-ray.
    3. You intubate the patient to allow for controlled mechanical ventilation.
    4. You apply a CPAP mask and initiate non-invasive positive pressure ventilation.
    5. You take an arterial blood sample for a blood gas analysis.

10. Which of the following medications are used in palliative care for the management of nausea and vomiting?

- - 1. Scopolamine, levomepromazine, ketamine, dronabinol
    2. Dexamethasone, haloperidol, etoricoxib, granisetron
    3. Dronabinol, tapentadol, scopolamine, haloperidol
    4. Levomepromazine, dexamethason, granisetron, ketamine
    5. Metoclopramide, levomepromazine, haloperidol, dronabinol

11. Which statement relating to delirium in palliative care is correct?

- - 1. Fluctuations during the course of delirium are atypical.
    2. A clear cause for delirium is found in most patients.
    3. In a non-convulsing patient, status epileptic us should be part of the differential diagnosis for a sudden onset delirium.
    4. The initial management for an agitated delirious patient includes benzodiazepines.
    5. Delirium invariably signals the beginning of the dying process.

12. Which of the following routes and/or combinations should never be used in the palliative medicine setting for medication delivery?

- - 1. A continuous subcutaneous mixture of Metamucil, haloperidol and hydromorphine
    2. Rectal insertion of slow-release morphine tablets
    3. Transdermal buscopan
    4. Topical application of (or wound irrigation with) a solution of ropivocaine and adrenaline
    5. Inhaled dronabinol

13. Which statement relating to palliative care patients is NOT correct?

- - 1. Intravenous fluid therapy in the dying phase can lead to an increase in pain, dyspnoea and nausea.
    2. Oxygen delivery can lead to distressing thirst.
    3. Distressing thirst can be always be eased through adequate mouth-care.
    4. The spiritual needs of the patient should NOT be addressed by the doctor, as patients find this very difficult to deal with.
    5. Regular airway suctioning in dying patients with a "death rattle" breathing pattern is seldom helpful.

14. Which statement is correct?

The "death rattle" heard in dying patients...

- - 1. Should preferably be managed with medication.
    2. Is very distressing for the patient.
    3. Should be in good time and prophylactically managed through fluid restriction.
    4. Is heard in nearly every patient.
    5. Can have its onset several days before a patients death.

15. Which statement is correct?

- 1. A high analgesic or sedative requirement to control distressing symptoms, accompanied by the acceptance of a hastened death (indirect active euthanasia) is illegal in Germany.
  2. Both the withdrawing active feeding via Nasogastric tube and withholding the initiation of nasogastric feeds would be considered as “allowing a patient to die” (passive euthanasia)
  3. Switching off a ventilator at the request of the patient with the consequence of inevitable death is considered to be “request killing” (voluntary active euthanasia)
  4. The acceptance of a potential shortening of life through medication given to control distressing symptoms is considered to be a voluntary active euthanasia.
  5. Stopping dialysis correlates to a so-called “medical end-of-life decision” (indirect active euthanasia)

16. A patient with a living will (advanced directive) clearly stated that under no circumstances is mechanical ventilation to be considered as a treatment option. The patient decompensated and was, incorrectly, intubated and mechanically ventilated, as the treating doctor was not aware of the existence of the existing directive). Now that the instructions in the advance directive have been confirmed, stopping mechanical ventilation would be considered….

- 1. Voluntary active euthanasia
  2. Passive euthanasia
  3. Direct active euthanasia
  4. Indirect active euthanasia
  5. Assisted Suicide

17. An 86 year old female patient is suspected of having an intra-cerebral haemorrhage after collapsing at home. New onset focal seizures are noted, most prominent in the face. You insert a nasogastric tube to allow for antiepiletic drug administration as well as enteral feeding (as the patient can no longer swallow). The patient improves somewhat over the next few says. During this time, the patient and family together produce a power of attorney and a living will (advance directive). In the living will, the patient explicitly refuses the insertion of a nasogastric tube for the purpose of enteral nutrition. The relatives request an end to this “cruel treatment” (through removal of the nasogastric tube) so that the patient may have a dignified death. You proceed as follows:

- 1. You immediately order the removal of the nasogastric tube and stop the enteral feeds in accordance with the living will
  2. You do NOT follow the living will, as it has not been executed by a lawyer and is thus not valid.
  3. You speak directly with the patient to clarify her wishes
  4. You explain to the relatives that, in accordance with medical ethics, you are not allowed to let the patient starve to death.
  5. You arrange for a court appointed legal representative to take over the matter

18. Which statement relating to living wills (advance directives) is most correct? The living will…

- 1. …is, like a standard will, required to be executed by a lawyer in order to be considered valid and binding
  2. …can include an order for phycision assisted suicide in the case of palliative care patients, and is binding.
  3. …is binding in the case of the patient no longer being unable to give consent
  4. … becomes null and void if a DNR (do not resuscitate) order is issued
  5. …cannot include instructions relating to organ donation, post-mortem examination or funeral plans.

19. A 53 year old female patient known with ovarian cancer is transferred to the Intensive Care Unit after a diagnostic laparotomy. The planned tumour resection was cancelled after discovery of peritoneal metastases during the operation. During the grand round (multidisciplinary ward round with different doctors, nurses allied health professionals etc) the following day, the patient asks if the operation went well. You respond as follows:

- 1. You immediately and truthfully inform the patient of the implications of peritoneal metastasis, and state the prognosis
  2. You continue with the grand round, and undertake to visit the patient afterwards to discuss the diagnosis in private
  3. You inform the patient that the case leader is the gynaecologist and you then inform the gynaecologist that the patient wishes to talk to him or her.
  4. You explain to the patient that the operation did not go as well as was expected, and you arrange an appointment to speak to her in more detail after the grand round.
  5. You explain to the patient simply that the diagnostic laparotomy is finished.

20. A 65 year old male patient with a complicated post operative course has been in your intensive care unit for several weeks. His condition is progressively deteriorating, and you are of the opinion that all medically indicated curative interventions have been exhausted. Due to the patients pain, dyspnoea and weakness, you do not consider a transfer to a normal ward to be appropriate. During the grand round however, the patient makes the express wish to “just go home” and to “not have to suffer anymore”. What is the most appropriate course of action?

- 1. You explain to the patient that comprehensive management in the intensive care unit is the best course, and that his wish to go home is unfortunately not possible.
  2. You request a psychiatry consultation, as you suspect the patient is having a major depressive episode.
  3. You calm the patient, and assure him that any worsening of his symptoms or any increase in his suffering with be treated with an overdose of pain medication.
  4. You suggest a family meeting in order to discuss the available options for palliative care treatments.
  5. You request a psychological consultation to help the patient to cope with his illness.
